# Dynamic Characteristics of Clay-Rubber Mixtures: Perspective on Small-Strain Dynamic Shear Modulus and Damping Ratio

**DOI:** 10.3390/ma19040780

**Published:** 2026-02-17

**Authors:** Bingheng Liu, Yong Wang, Jianqun Zhu, Guofang Xu

**Affiliations:** 1 School of Civil Engineering and Architecture, Changzhou Institute of Technology, Changzhou 213032, China; zhu-jq@163.com; 2 State Key Laboratory of Geomechanics and Geotechnical Engineering, Institute of Rock and Soil Mechanics, Chinese Academy of Sciences, Wuhan 430071, China; wangyong@whrsm.ac.cn (Y.W.); gfxu@whrsm.ac.cn (G.X.)

**Keywords:** clay-rubber mixtures, small-strain, dynamic shear modulus, damping ratio, Hardin–Drnevich equation, prediction of maximum dynamic shear modulus

## Abstract

Waste tire rubber–soil mixtures feature low density, high energy dissipation, and low shear modulus, which are widely used in geotechnical engineering for vibration attenuation. In this study, the evolution of the small-strain stiffness characteristics of clay-rubber mixture (CRM) is investigated; a resonance column test was carried out to determine the small-strain stiffness characteristics of CRM samples with different confining pressures ($\sigma_{3}$), rubber particle contents ($C_{\mathrm{rubber}}$), and rubber particle sizes ($D_{\mathrm{rubber}}$). The test results indicate that $\sigma_{3}$ can promote the dynamic shear modulus (*G*) of CRM and restrain the damping ratio (*D*). The rubber particles have a great influence on both *G* and *D*. Under the same conditions, *G* decreases significantly with the increase in $C_{\mathrm{rubber}}$ and increases slightly with the increase in $D_{\mathrm{rubber}}$, which indicates that rubber particles inhibit the development of *G*. *D* increases with the increase in $C_{\mathrm{rubber}}$ and $D_{\mathrm{rubber}}$. The results show that the contact area between clay particles and rubber particles increases with the increase in $C_{\mathrm{rubber}}$, resulting in the decreases in *G* and *D*. The *G–γ* curves are analyzed by using the Hardin–Drnevich equation. Based on the fitting results, the maximum dynamic shear modulus ($G_{\max}$) is obtained. Therefore, the evolution of $G_{\max}$ with $\sigma_{3}$, $C_{\mathrm{rubber}}$, and $D_{\mathrm{rubber}}$ are analyzed, and an equation for the $G_{\max}$ of CRM considering the effects of $\sigma_{3}$, $C_{\mathrm{rubber}}$, and $D_{\mathrm{rubber}}$ is proposed. In addition, the *D–γ* curves can be well described by an empirical equation.

## 1. Introduction

With the improvement in people’s living standards and the rapid development of the automobile industry, the number of vehicles around the world continues to grow. As a result, the number of waste tires is increasing day by day [1,2,3,4,5,6,7]. The accompanying series of problems in the disposal of waste rubber tires is also something that people must face and solve. The traditional methods of disposing of waste rubber tires mainly include the following four schemes: “landfilling”, “hoarding”, “incineration”, and “decomposition and recycling” [8,9,10,11,12]. Nevertheless, all the aforementioned treatment approaches for waste rubber tires possess certain limitations, exerting an influence on the treatment outcome. For instance, “landfilling” may affect the ecological environment, “hoarding” occupies a considerable amount of land resources, and “incineration” may release a lot of harmful gases that seriously threaten the environment and human health; regarding the “decomposition and recycling” of waste rubber tires, the scale is relatively small and the recycling outcome is not yet satisfactory [13,14]. As a result, the question of how to rationally handle waste rubber tires has been perplexing the relevant practitioners and engineers. At present, waste rubber tires can be recycled and utilized as modified materials for soil. Waste rubber tires can also be crushed and processed as engineering materials to be applied to civil engineering (the metal and fabric components therein are eliminated), which can meet the large demand for materials for engineering construction [1,2,3]. In addition, it can meet the requirements of environmental protection and produce certain economic benefits.

In geotechnical engineering, waste rubber tires are often crushed into rubber particles (the metal and fabric components therein are eliminated), which are then mixed with the soil. Due to the poor static and dynamic properties of many soils in practical engineering, appropriate treatments are necessary to ensure stability and safety [15,16,17]. The soil-rubber mixtures have the characteristics of low density, high elasticity, high energy consumption, and low shear modulus, which are widely used in soft soil foundation treatment, retaining walls, thermal insulation materials, buried pipes, and other geotechnical engineering, can improve the mechanical properties of soil and play a role in vibration isolation and vibration reduction [3,18,19,20]. In particular, rubber–soil mixture has been extensively applied in the design of shock absorption [21,22,23]. As important dynamic properties of soil, the dynamic shear modulus (*G*) and damping ratio (*D*) are very important for calculating the deformation of foundations in practical engineering [24,25,26].

Many studies have reported on the evolution of the small-strain stiffness and damping ratio of rubber–soil mixtures and pointed out that the rubber has a significant effect on the dynamic properties of the mixtures [27,28,29,30]. Feng and Sutter [31] conducted resonance column tests to obtain the variation in *G* and *D* of the mixtures with rubber particle content and argued that the maximum dynamic shear modulus could be predicted by the proposed model. Edincliler et al. [32] studied the factors that affect the dynamic shear modulus of sand-rubber mixtures with different rubber shapes and contents by cyclic triaxial tests. Nakhaei et al. [33] conducted consolidated undrained cyclic triaxial tests to study the influence of rubber particle content on the *G* and *D* of rubber–sand mixtures and found that both the *G* and *D* of rubber–sand mixtures decreased with increasing rubber particle content. Pistolas et al. [34] investigated the effects of rubber particle content and average particle size of rubber particles on the *G* and *D* of rubber–sand mixtures through resonance column and cyclic triaxial tests. The results indicated that with the increase in rubber particle content, the *G* of the mixtures decreased, while the *D* increased. Senetakis et al. [35] proposed the curves of normalized *G* and *D* versus shear strain for sand-rubber and gravel-rubber mixtures using resonance column tests and derived the empirical formulas for the *G* and *D* of rubber–sand mixtures. Some scholars have also utilized dynamic triaxial tests to analyze the developments of shear modulus and damping ratio (from small to large strains) in sand–rubber particle mixtures with respect to rubber content and morphology and have proposed feasible recommendations for improving the seismic isolation properties of sand [36,37]. In summary, an increase in rubber content inhibits the development of *G* in rubber–soil mixtures. In addition, rubber particle size has a non-negligible impact on the *G* and *D* of rubber–soil mixtures [38,39,40]. For example, Xia et al. [41] investigated the influence of rubber particle size on the *G* and *D* of rubber–clay mixtures through resonance column tests. It was discovered that the variation in *G* was the same as that of the pure clay. Both the *G* and *D* of the rubber–clay mixtures decreased with the increase in rubber particle size.

Overall, there have been relatively few studies on the evolutions of the *G* and *D* of rubber–clay mixtures, and the influence of rubber particle size on the small-strain stiffness has been less investigated. Prior research has predominantly focused on sandy soils, silts, or high-plasticity clays, often employing relatively large rubber particles (e.g., diameters > 0.2 mm). Furthermore, systematic studies aiming to quantify the small-strain stiffness of clay-rubber mixtures and develop corresponding predictive formulations remain limited. Therefore, the effects of both the rubber particle content and rubber particle size of the clay-rubber mixtures should be considered in the investigation. The maximum shear modulus ($G_{\max}$), i.e., the initial shear modulus, under very small-strain conditions is another aspect of the study of the small-strain dynamic characteristics of soils. There are many factors affecting $G_{\max}$, such as confining pressure, pore ratio, etc. The famous Hardin–Drnevich equation is often used to calculate $G_{\max}$ of soil [42], which can reflect the influences of over-consolidation ratio, pore ratio, and effective stress on $G_{\max}$. However, the applicability of this formula to the clay-rubber mixtures needs to be further studied.

In this study, a large number of resonance column tests were conducted on the clay-rubber mixtures (CRMs). The influences of particle sizes and contents of waste rubber particles on the evolution of the small-strain dynamic shear modulus and damping ratio of CRM under various confining pressures were analyzed. The test results were analyzed, and it was discovered that the Hardin–Drnevich model was relatively suitable for fitting the *G–γ* curves. Finally, in accordance with the fitting results, an empirical model for predicting the maximum shear modulus of CRM was estimated, considering the effects of the particle sizes, contents of waste rubber particles, and confining pressure.

## 2. Materials and Methods

### 2.1. Materials and Sample Preparations

**Figure 1:**
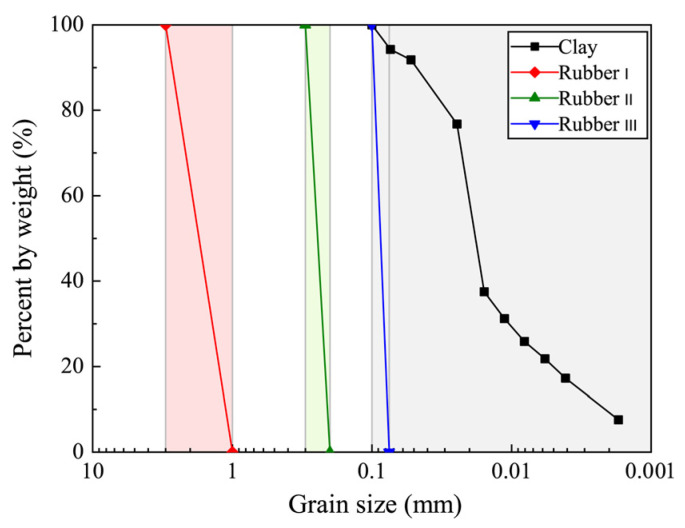
Characteristics of rubber particle and clay particle size distribution.

In this work, the sample was a mixture of rubber and clay particles. The rubber particles used in this study were obtained from a rubber tire recycling plant (the metal and fabric components therein were eliminated), with average particle sizes ($D_{\mathrm{rubber}}$) of 0.085 mm, 0.25 mm, and 2 mm, and the particle size distributions are shown in Figure 1 (Rubber I, Rubber II, and Rubber III in Figure 1). The properties of the clay used in this study are presented as follows: the water content was 23.4%, the specific gravity of the clay particles was 2.68, and the liquid limit and plastic limit of the clay were 40.2% and 17.6%, respectively. In accordance with the USCS classification method [43], this clay can be classified as CL. CL clay refers to clay soils classified as “CL”, meaning low to medium plasticity clay. Comparing the particle size distribution curves of rubber and clay particles (Figure 1), it is obvious that the average particle size of rubber particles is larger than that of clay particles. Due to the fact that there are relatively few studies on the small-strain characteristics of clay under the influences of rubber particle content and average particle size, it is necessary to prepare samples of clay-rubber mixtures with various rubber particle sizes and contents.

**Figure 2:**
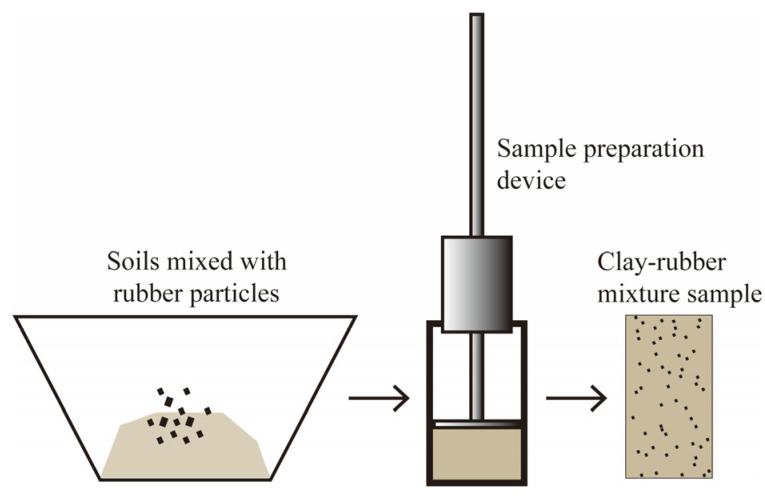
The fabrication methods and processes of clay-rubber mixture cylinder samples.

The CRM sample was cylindrical, with a diameter of 50 mm and a height of 100 mm. The samples of clay-rubber mixtures with a rubber particle size of 0.25 mm and rubber particle content of 5%, 10% and 15% and the samples with a rubber particle content of 15% and rubber particle sizes of 0.085 mm, 0.25 mm and 2.0 mm were prepared, respectively. Rubber particle content refers to the ratio of rubber particle volume to the volume of dry clay. The method for preparing the clay-rubber mixture sample is shown in Figure 2. The specimen preparation followed the Chinese standard for geotechnical testing methods (GB/T 50123-2019) [44]. The required amount of water was sprayed onto the clay and mixed with rubber particles thoroughly. The mixture was placed in a plastic bag and stored in a sealed container for at least 20 h to allow uniform moisture distribution. Following this equilibration period, the water content was re-measured. Prior to compaction, the inner diameter of the compaction mold was selected to match the specimen diameter. A compaction hammer with a diameter slightly smaller than that of the specimen was preferred, although a hammer of equal diameter was also acceptable. The inner surface of the mold was cleaned, wiped, and lightly coated with a thin layer of vaseline before use. The CRM required for the specimen was calculated and weighed according to the target dry density. The specimen was compacted in layers corresponding to its height, and it was compacted in 3 layers. An equal mass of CRM was used for each layer. After each layer was compacted to the specified height, its surface was scarified before placing the CRM for the subsequent layer. This process was repeated until the final layer was compacted. Both ends of the specimen in the compaction mold were trimmed flat, after which the specimen was then prepared.

While the preparation method may induce a degree of anisotropy, this effect is systematic and uniform across all specimens. All samples were prepared following the same procedure. As a result, the observed trends can be attributed to variations in material composition, rather than to inconsistencies in how the samples were made.

### 2.2. Test Apparatus

**Figure 3:**
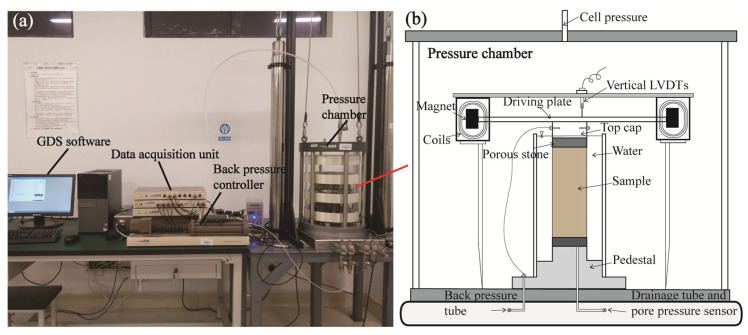
The layout of the resonance column equipment: (**a**) photograph of the device; (**b**) the schematic diagram of the pressure chamber.

The equipment used in this study was the Stoke resonance column apparatus (RCA) produced by GDS Instruments Pte. Ltd. (Hook, UK), and its photograph is shown in Figure 3a. The RCA is composed of control software, a data acquisition unit, a back pressure controller, a pressure chamber, and an air compressor. In this study, a sample with a diameter of 50 mm and a length of 100 mm was used, which was fixed on the base plate of the RCA (Figure 3b). A device for twisting and bending vibrations was placed on the top of sample. The excitation mode could be re-set as per the requirements of the software, enabling torsional and bending vibration tests to be conducted under the same conditions. The measurement range of the vertical displacement sensor is 100 mm, the maximum axial dynamic load frequency is 70 Hz, and the pressure chamber is a standard pressure chamber with a maximum air pressure of 1.0 MPa.

### 2.3. Test Procedure and Test Program

Prior to resonant column testing, the specimens were saturated using a standard back pressure saturation procedure. After specimen installation, a low effective confining pressure was applied, and both cell pressure and back pressure were increased incrementally while maintaining a nearly constant effective stress. At each increment, sufficient time was allowed for pore pressure equalization. The degree of saturation was evaluated using the *B*-value, which was measured by applying a small increase in cell pressure and recording the corresponding pore pressure response. The saturation process was continued until the *B*-value of each specimen exceeded 0.97, indicating a fully saturated condition suitable for resonant column testing.

After the sample was saturated, it was installed on the RCA, then the top cap was placed on its upper end. Subsequently, the driving system of the top cap was mounted onto the sample, and a tight connection between the driving disk and the sample was maintained in the tests. The accelerometer and drainage lines were installed, and the LVDTs were attached, as shown in Figure 3b. Then, the consolidation process was carried out. The consolidation pressures were set as 50 kPa, 100 kPa, 200 kPa, 300 kPa, and 400 kPa, respectively. After consolidation, resonance column tests were carried out on the sample to obtain the dynamic shear modulus (*G*) and damping ratio (*D*) of the sample under small-strain conditions (shear strain of 0.001%–0.1%). Table 1 shows the test scheme. The particle size of the rubber was selected to be comparable to that of the clay particles, with the primary objective of investigating the influence of clay-sized rubber particles on the small-strain shear stiffness of clay-rubber mixtures (CRMs). This choice allows the interaction between rubber particles and the clay matrix to be examined without introducing significant fabric changes associated with coarse inclusions. The relatively low rubber contents were considered to explore whether the damping characteristics of clay could be enhanced while minimizing rubber consumption, thereby improving material efficiency and practical applicability.

For the measurement of the shear modulus, sinusoidal excitation was generated by an electromagnetic driving system. The system is composed of a four-arm rotor and a support column. Concretely, a permanent magnet is affixed to the bottom of each arm, and four pairs of coils are fixed to the support column. A sinusoidal voltage was applied to the coils, and a torque was thereby induced in the sample. The frequency and amplitude of the applied voltage were varied until the resonance frequency of the sample was identified. The shear modulus *G* was subsequently calculated by the following equation:

(1) $$G\;=\;\rho {(2\pi \mathrm{fH}/\beta )}^{2}$$

where *ρ* is the density of the sample; *f* is the frequency; *H* is the height of the soil sample; and *β* is the eigenvalue of the frequency equation.

Upon completion of the torsional resonance test, the resonance frequency of the sample was obtained. The test mode was automatically switched to the damping test, and the resonance frequency was adopted as the input parameter for damping evaluation. The damping ratio *D* was determined from the free-vibration decay curve, which was recorded by an accelerometer on the driving disk. A sinusoidal wave was applied to the sample, the excitation was abruptly terminated, and the resulting free vibration was measured. The value of the damping ratio can be calculated by solving Equation (2).

(2) $$D=\sqrt{\frac{\delta^{2}}{4\pi^{2}+\delta^{2}}}$$

where *δ* is the logarithmic decrement.

## 3. Results and Discussion

### 3.1. Evolution of Shear Modulus

**Figure 4:**
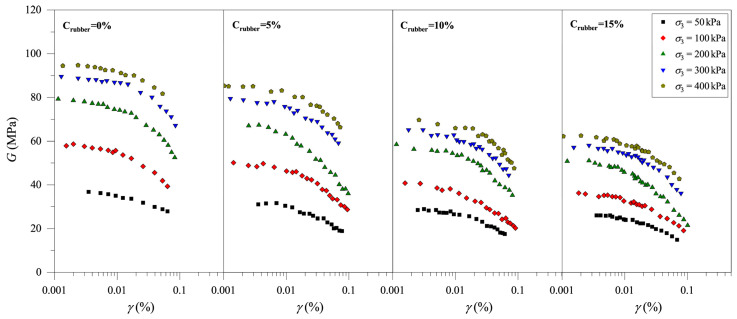
Evolution of shear modulus of clay-rubber mixture with different rubber particle contents.

According to the results of the resonance column test, it is found that rubber particle content, rubber particle size, and consolidation pressure all have significant effects on the *G*–*γ* curves of CRM. Briefly, the rubber particle content is represented by $C_{\mathrm{rubber}}$, the rubber particle size is represented by $D_{\mathrm{rubber}}$, the consolidation pressure is represented by $\sigma_{3}$, and the shear strain is represented by *γ*. Figure 4 shows the attenuation of *G* with the *γ* of CRM for different rubber particle contents ($C_{\mathrm{rubber}}$ = 0%, 5%, 10%, and 15%; $D_{\mathrm{rubber}}$ = 0.25 mm) and different consolidation pressures ($\sigma_{3}$ = 50 kPa, 100 kPa, 200 kPa, 300 kPa, and 400 kPa). The values of *G* decrease with increasing *γ*, and the decreasing rate of *G* increases as *γ* increases. In addition, for all samples, the value of *G* increases with the increase in $\sigma_{3}$ at the same *γ* value. That is, under the same conditions, the value of *G* increases with the increase in stress level, which is similar to the resonance column test results of the pure clay. With the same value of *γ*, the value of *G* under a high value of $\sigma_{3}$ is higher than that under a low value of $\sigma_{3}$. This is the concrete embodiment of the high stiffness of the soil under high consolidation pressure. With the increase in stress levels, the pore volume in CRM decreases and the connection between particles in the CRM becomes closer, resulting in a significant increase in the stiffness of the CRM. By comparing the *G–γ* curve of the CRM with different $C_{\mathrm{rubber}}$, it can be found that the value of *G* decreases with increasing $C_{\mathrm{rubber}}$. The above experimental results indicate that rubber particles reduce the stiffness of the CRM under the same conditions, which may be due to the low shear resistance of rubber particles.

**Figure 5:**
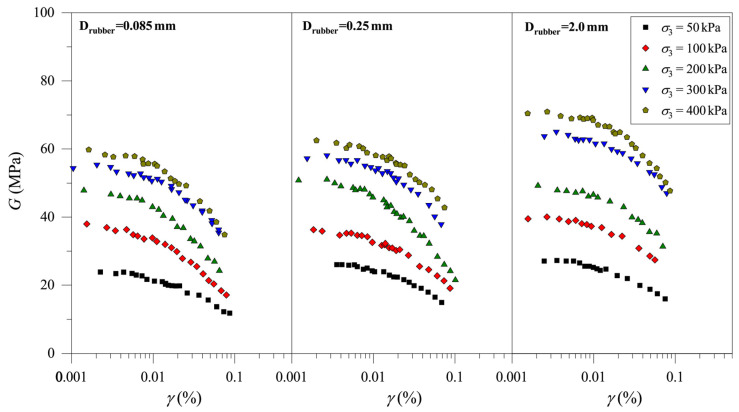
Evolution of the shear modulus of the clay-rubber mixture with different rubber particle sizes.

Figure 5 shows the *G–γ* curves of the mixtures with different rubber particle sizes ($C_{\mathrm{rubber}}$ = 15%; $D_{\mathrm{rubber}}$ = 0.085 mm, 0.25 mm, and 2.0 mm) and different consolidation pressures ($\sigma_{3}$ = 50 kPa, 100 kPa, 200 kPa, 300 kPa, and 400 kPa). Similarly to the test results in Figure 4, the value of *G* increases with increasing $\sigma_{3}$. In addition, under the same values of $\sigma_{3}$ and $C_{\mathrm{rubber}}$, the value of $D_{\mathrm{rubber}}$ has a significant effect on the value of *G*. The value of *G* shows a slight increasing trend with increasing $D_{\mathrm{rubber}}$. The test results will be further analyzed in the following section.

### 3.2. Evolution of Damping Ratio

Damping ratio (*D*) is an important dynamic parameter that characterizes the dynamic hysteresis and energy dissipation of soil, which has been well studied in scientific research and practical engineering. In this study, the development of *D* at different shear strains is obtained from the attenuation characteristics of dynamic parameters. Similarly to the evolution of *G*, *D* is affected by the coupling of rubber particle content, rubber particle size, stress level, and shear strain.

**Figure 6:**
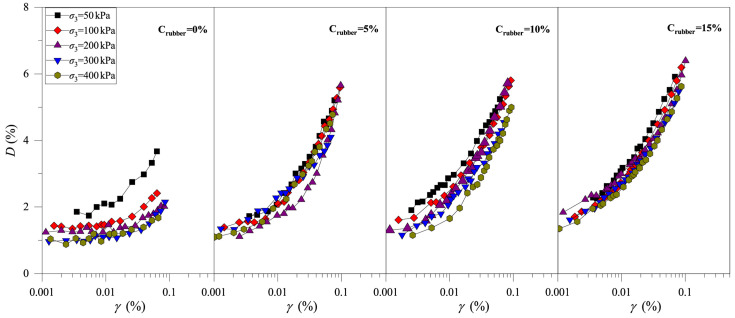
Development of the *D–γ* curve of the clay-rubber mixture with different rubber particle contents.

Figure 6 show the evolutions of the *D–γ* curves of CRM for different rubber particle contents ($C_{\mathrm{rubber}}$ = 0%, 5%, 10%, and 15%; $D_{\mathrm{rubber}}$ = 0.25 mm) and different consolidation pressures ($\sigma_{3}$ = 50 kPa, 100 kPa, 200 kPa, 300 kPa, and 400 kPa). It can be observed that, under the same conditions, the *D* values of the clay and CRM samples increase with the increase in shear strain, indicating that the shape of the *D–γ* curve is not significantly affected by $C_{\mathrm{rubber}}$. For CRMs with different $C_{\mathrm{rubber}}$, the value of *D* decreases with the increase in stress level, which indicates that an increase in stress level reduces the dynamic hysteresis characteristics of CRM. On the whole, *D* increases slightly with increasing $C_{\mathrm{rubber}}$. The above test results show that the rubber particles strengthen the dynamic hysteresis characteristics of CRM. That is, the rubber particles can enhance the ability of energy absorption for CRM.

**Figure 7:**
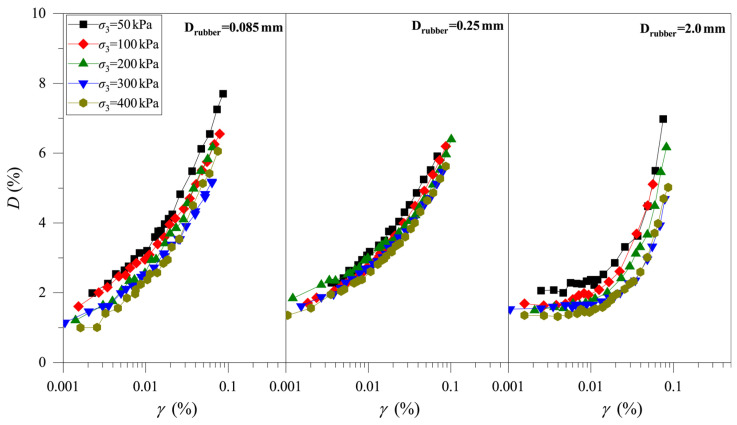
Development of the *D–γ* curve of the clay-rubber mixture under different rubber particle sizes.

Figure 7 shows the *D–γ* curves of CRM under different rubber particle sizes ($C_{\mathrm{rubber}}$ = 15%; $D_{\mathrm{rubber}}$ = 0.085 mm, 0.25 mm, and 2.0 mm) and different stress levels ($\sigma_{3}$ = 50 kPa, 100 kPa, 200 kPa, 300 kPa, and 400 kPa). Like the test results in Figure 6, the *D* of CRM under the influence of different $D_{\mathrm{rubber}}$ also increases with increasing stress level, and the value of *D* also increases with increasing shear strain. Under the same conditions, the values of *D* show a slight increase with the increase in $D_{\mathrm{rubber}}$. Consequently, to maintain the damping capacity of mixtures, the use of larger rubber particles is recommended for clay modification.

### 3.3. Mechanism Analysis of the Influence of Rubber Content and Particle Size on the Small-Strain Modulus of Clay-Rubber Mixtures

CRM is a type of mixture of rubber particles and clay. In general, the volume of rubber particles does not change significantly during the consolidation process. Previous studies indicated that rubber could not resist shear deformation caused by vibration [11]. Based on the physical and mechanical properties of rubber particles, the rubber particles in CRM can be regarded as the pores in CRM. Given that there are two kinds of solids in CRM, the value of the pore ratio (*e*) is difficult to determine. The parameter of equivalent pore ratio ($e_{\mathrm{eq}}$) can be introduced to reflect the pore characteristics of CRM. The definition of $e_{\mathrm{eq}}$ is shown in Equation (3) [45]:

(3) $$e_{\mathrm{eq}}=\frac{V_{\mathrm{void}}+V_{\mathrm{rubber}}}{V_{\mathrm{clay}}}$$

where $V_{\mathrm{void}}$, $V_{\mathrm{rubber}}$, and $V_{\mathrm{clay}}$ are the pore volume of clay, volume of rubber particles, and volume of clay particles, respectively. When $V_{\mathrm{rubber}}$ increases, the pore volume of CRM increases and the small-strain stiffness decreases. Therefore, the dynamic shear modulus of CRM decreases with increasing $C_{\mathrm{rubber}}$.

**Figure 8:**
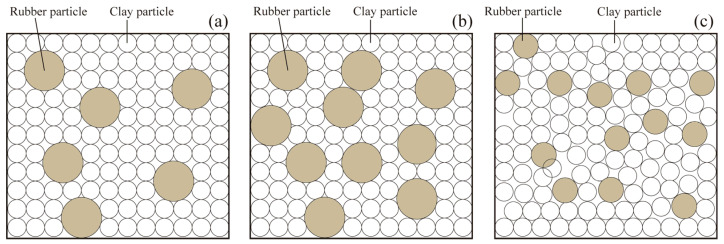
Schematic diagram of distribution of clay particles and rubber particles: (**a**) large $D_{\mathrm{rubber}}$ with small $C_{\mathrm{rubber}}$; (**b**) large $D_{\mathrm{rubber}}$ with large $C_{\mathrm{rubber}}$; (**c**) small $D_{\mathrm{rubber}}$ with small $C_{\mathrm{rubber}}$.

Compared to clay particles, rubber particles possess a notably lower elastic modulus and reduced shear modulus. For CRMs with different $D_{\mathrm{rubber}}$, *G* increases slightly with the increase in $D_{\mathrm{rubber}}$. It may be assumed that rubber particles are evenly distributed in $D_{\mathrm{rubber}}$, and the schematic diagrams of rubber particles and clay particles in CRM are shown in Figure 8. When the total weight of rubber is kept constant, smaller particles result in a higher specific surface area and a larger particle count. Therefore, the surface area of rubber particles increases, resulting in an increase in the contact area between the clay particles and rubber particles in CRM. When the particle size of rubber particles increases, the specific surface area of the rubber particles decreases significantly, so the total surface area of rubber particles decreases. This results in a decrease in the contact area between clay particles and rubber particles in CRM. Because the *G* of clay particles is higher than that of rubber particles, the *G* of CRM with a larger contact area between clay particles and rubber particles is smaller. As a result, with the increase in rubber particle diameter, the *G* of CRM shows a slight increasing trend.

### 3.4. Characteristics of Dynamic Shear Modulus of Clay-Rubber Mixture

The characteristics of the *G–γ* curve of CRM are analyzed in this section. The characteristics of the *G–γ* curve of CRM are similar to those of pure clay and sand, so the fitting curve commonly used for clay and sand can be used to describe the dynamic shear modulus of CRM. Generally, the stress–strain curve of soil under dynamic loading is obtained by using equivalent linear analysis. Based on the Masing criterion, Hardin and Drnevich [42] describe the relationship between *G* and *γ* in a resonance column test and cyclic simple shear test using the following equations:

(4) $$G(\gamma )\;=\;G_{\max}(1-f(\gamma ))$$

(5) $$f(\gamma )\;=\frac{\gamma /\gamma_{\mathrm{ref}}}{1\;+\;\gamma /\gamma_{\mathrm{ref}}}$$

where *G*(*γ*) is the dynamic shear modulus of soil when the shear strain is *γ*; $G_{\max}$ is the maximum dynamic shear modulus of soil (initial shear modulus), which represents the shear modulus of soil when the shear strain is 0; and $\gamma_{\mathrm{ref}}$ is the reference shear strain. The physical meaning of $\gamma_{\mathrm{ref}}$ is the ratio of the maximum shear stress to the initial shear modulus within the hysteresis loop during cyclic loading. The reference shear strain, as defined within this study, is the strain parameter used to normalize the applied strain in order to more simply and consistently define small-strain behaviors under varying conditions. By integrating the above two equations, the Hardin–Drnevich equation can be obtained as follows:

(6) $$G(\gamma )\;=\;G_{\max}\frac{1}{1\;+\;\gamma /\gamma_{\mathrm{ref}}}$$

Some scholars have revised this formula in order to improve the simulation effect of the formula [46,47,48]. The three-parameter Martin–Davidenkov model improved by Martin and Seed [48] has a wide application. This formula is appropriately modified on the Hardin–Drnevich equation:

(7) $$G(\gamma )\;=\;G_{\max}{(1-\frac{{\left(\gamma /\gamma_{0}\right)}^{2B}}{{1\;+\;(\gamma /\gamma_{0})}^{2B}})}^{A}$$

where $\gamma_{0}$, *A*, and *B* are parameters related to soil properties, but *γ*_0_ has no significant physical meaning.

**Figure 9:**
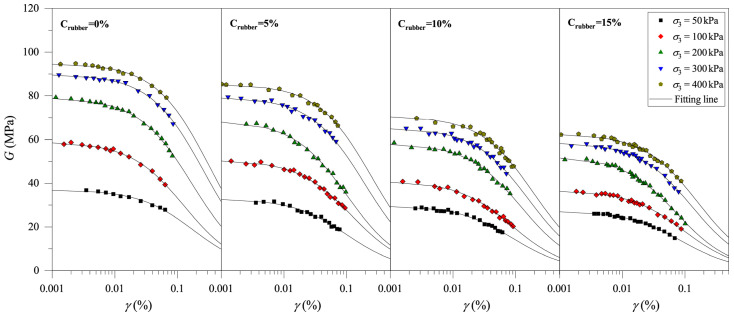
Fitting results of *G–γ* curve of CRM with different rubber particle contents.

**Figure 10:**
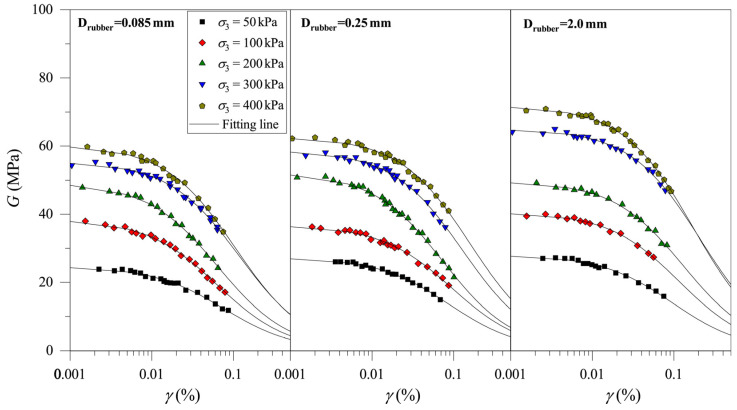
Fitting results of *G–γ* curve of CRM with different rubber particle sizes.

The primary rationale for ultimately selecting the Hardin–Drnevich model was not the inferior statistical performance of the Martin–Davidenkov model, but rather the principle of model parsimony. Although both models provided a good fit, the Martin–Davidenkov model requires more parameters. For this study, the simpler Hardin–Drnevich model, with fewer parameters, was considered sufficient and more appropriate. It effectively captures the essential modulus degradation trend, thereby avoiding the use of the more complex Martin–Davidenkov model. Therefore, the *G–γ* curve of CRM is analyzed by Equation (6) in this study. After analysis, it is found that this formula is suitable for describing the *G–γ* attenuation curve of CRM, and the specific fitting results are shown in Figure 9 and Figure 10. Obviously, the fitting results are good, indicating that Equation (6) can reflect the influences of $C_{\mathrm{rubber}}$ and $D_{\mathrm{rubber}}$ on the development of *G* with the *γ* of CRM.

### 3.5. Maximum Dynamic Shear Modulus of Clay-Rubber Mixture

**Figure 11:**
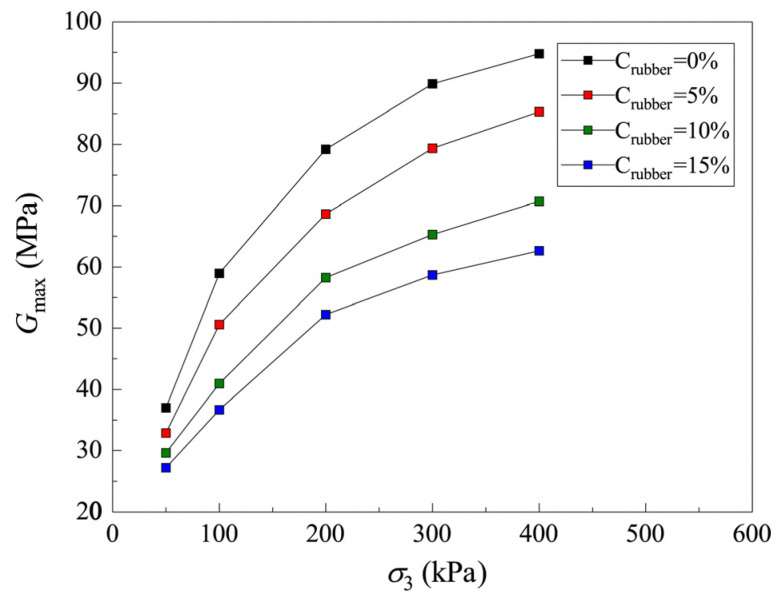
Relationship between $G_{\max}$ and $\sigma_{3}$ of CRM with different rubber particle contents.

The fitting results of the *G–γ* curves of CRM with $D_{\mathrm{rubber}}$ = 0.25 mm under different stress levels are analyzed, and the values of $G_{\max}$ and $\gamma_{\mathrm{ref}}$ are shown in Table 2. In order to directly observe the development of $G_{\max}$ with stress level, the relationship curves between $G_{\max}$ and $\sigma_{3}$ are shown in Figure 11 and the detailed parameters are shown in Table 2.

By observing the development of the value of $G_{\max}$ with the stress level, it is found that the relationship between $G_{\max}$ and $\sigma_{3}$ can be represented by a power function [49]:

(8) $$G_{\max}\;=\;{a\sigma_{3}}^{b}$$

where both *a* and *b* are fitting parameters. $G_{\max}$ values under all stress levels are fitted by using Equation (8), and fitting parameters *a* and *b* under different $C_{\mathrm{rubber}}$ can be obtained.

**Figure 12:**
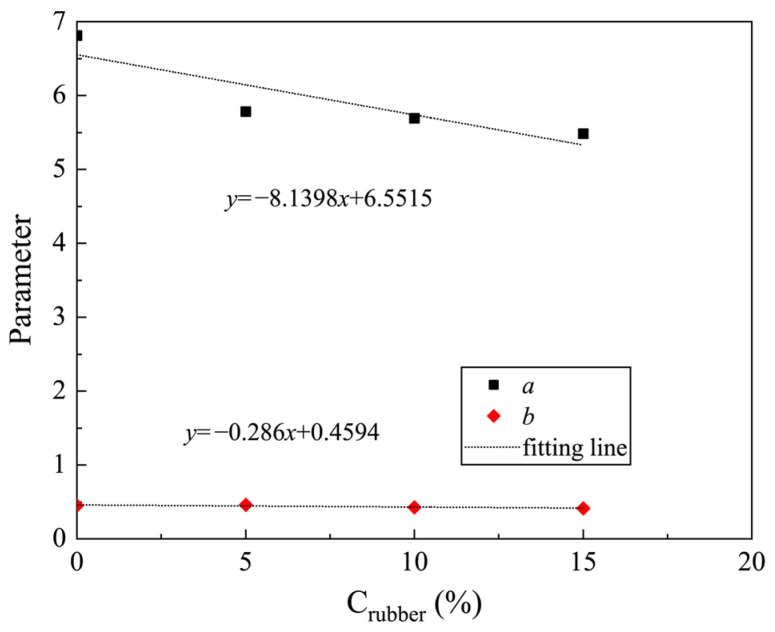
Relationship between fitting parameters *a* and *b* and rubber particle content of clay-rubber mixture.

To study the relationship of fitting parameters *a* and *b* with $C_{\mathrm{rubber}}$, the curves of *a* versus $C_{\mathrm{rubber}}$ and *b* versus $C_{\mathrm{rubber}}$ are plotted in Figure 12. It can be seen that the curves of *a–*$C_{\mathrm{rubber}}$ and *b–*$C_{\mathrm{rubber}}$ can be subscribed by linear equations. For convenience, the fitted linear equations are written directly in Figure 12. As confining pressure increases, the contact between soil and rubber within the CRM becomes closer. Consequently, with a higher rubber content, the shear modulus of the CRM under the same confining pressure decreases accordingly. Therefore, *b* decreases with an increase in $C_{\mathrm{rubber}}$.

By substituting the two linear equations in Figure 12 into Equation (8), a model for predicting $G_{\max}$ considering the consolidation pressure and rubber particle content can be obtained:

(9) $$G_{\max}\;=\;(-8.1398C_{\mathrm{rubber}}+6.5515){\sigma_{3}}^{-0.286C_{\mathrm{rubber}}+0.4594}$$

The fitting results of the *G–γ* curves of CRM with $C_{\mathrm{rubber}}$ = 15% and various $D_{\mathrm{rubber}}$ under different stress levels are shown in Table 3. It is evident that Equation (9), which reflects the small-strain shear modulus of CRM, has a specific range of applicability: the soil type must be CL clay with relatively fine particles (diameter < 0.1 mm), and the rubber particles should also be fine (diameter between 0.085 and 2 mm).

**Figure 13:**
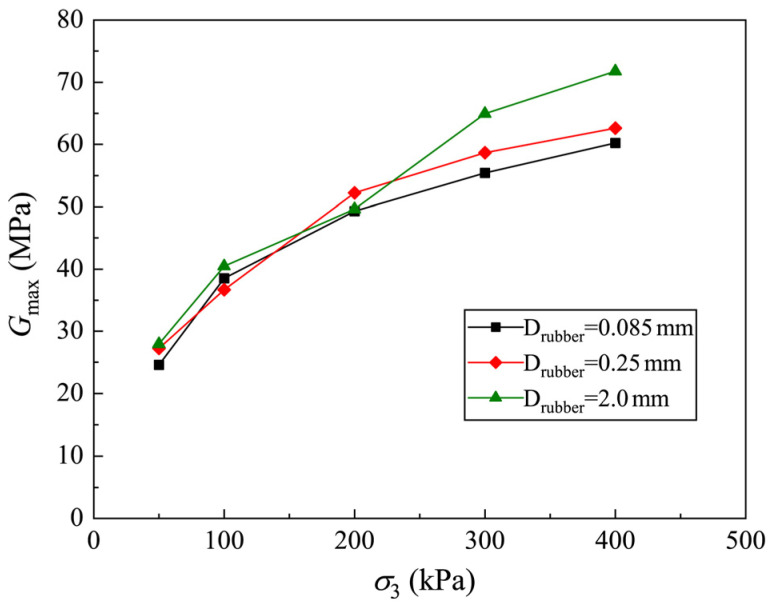
Relationship between $G_{\max}$ and $\sigma_{3}$ of clay-rubber mixtures with different rubber particle sizes.

In order to analyze the development of $G_{\max}$ with stress level for CRMs with different $D_{\mathrm{rubber}}$, the curves of $G_{\max}$ versus $\sigma_{3}$ are plotted in Figure 13. It is found that the fitting parameters of CRMs with different $D_{\mathrm{rubber}}$ are similar, and the difference in $G_{\max}$ under the same $\sigma_{3}$ is not large. In order to facilitate the application of the proposed model (Equation (9)), it can be considered that $D_{\mathrm{rubber}}$ has no influence on $G_{\max}$. The $G_{\max}$ of CRM under various $C_{\mathrm{rubber}}$ and $D_{\mathrm{rubber}}$ can be well described by Equation (9).

**Figure 14:**
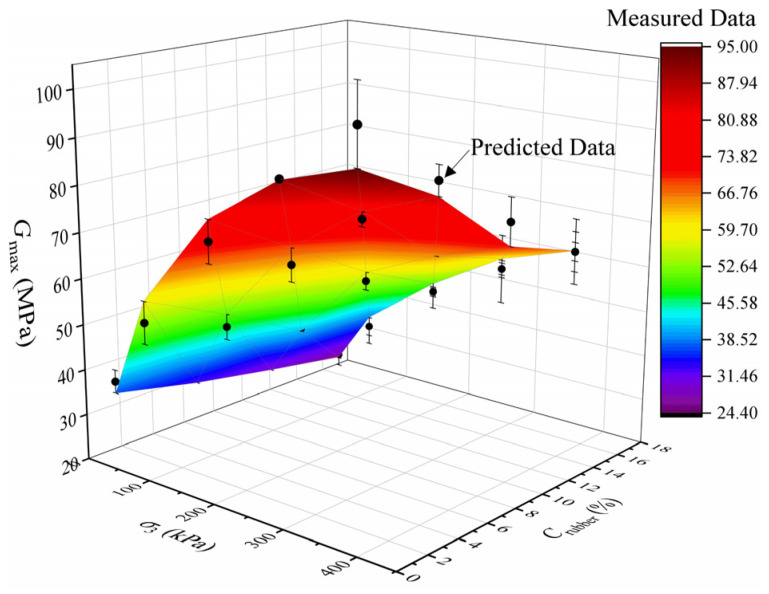
The contrastive results between the data calculated using the proposed model and the data acquired from experiments.

In order to verify the applicability of Equation (9), all the experimental data in this study are fitted by using this model, and the comparisons between the measured data and predicted data are plotted in Figure 14. It can be observed that the degree of coincidence between the experimental data and the predicted data is relatively high, demonstrating that the applicability of Equation (9) is satisfactory. Overall, the proposed model provides a novel perspective on understanding the dynamic shear modulus characteristics of clay-rubber mixtures. The research findings in this study offer theoretical and technical supports for enhancing the recycling and utilization of waste rubber tires in practical geotechnical engineering.

### 3.6. Fitting of the D–γ Curves of Clay-Rubber Mixture

Based on the development of dynamic shear modulus, Hardin and Drnevich proposed the following empirical formula for calculating the damping ratio [42]:

(10) $$D\;=\;D_{\max}(1-\frac{G}{G_{\max}})$$

where $D_{\max}$ is the maximum damping ratio; when *G* = 0, *D* = $D_{\max}$.

In engineering practice, since the Hardin–Drnevich hyperbolic model does not provide an ideal fit to the experimental data, the following empirical formula is commonly used [50]:

(11) $$D\;=\;D_{\max}{(1-\frac{G}{G_{\max}})}^{n}$$

where *n* is a fitting parameter related to the properties of the materials.

**Figure 15:**
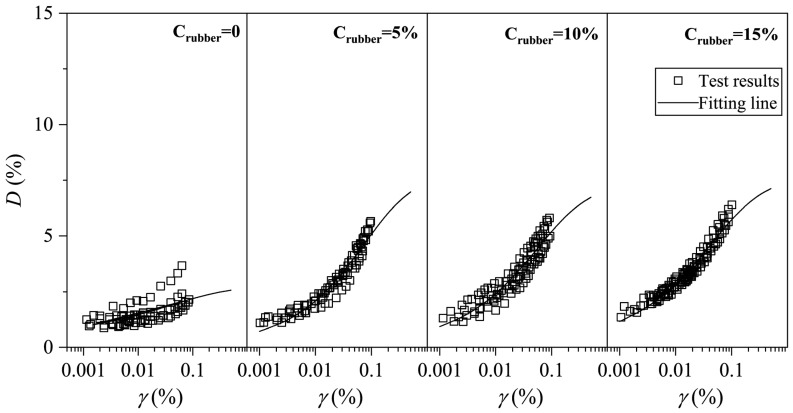
Fitting results of *D–γ* curve of CRM with different rubber particle contents.

**Figure 16:**
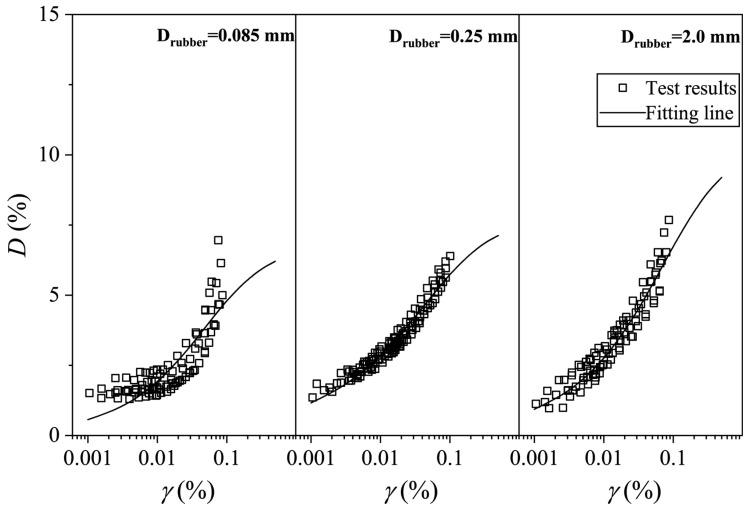
Fitting results of *D–γ* curve of CRM with different rubber particle sizes.

The experimental data points for mixtures under different consolidation pressures show minimal scatter and a narrow distribution band. The distribution of experimental data points for mixtures under different consolidation confining pressures and the model fitting curves are shown in Figure 15 and Figure 16. The analysis indicates that the selected empirical formula for the variation in damping ratio with strain can effectively capture the development of *D* with *γ*.

According to the fitting results of the *D–γ* curve, the confining pressure has little influence on the fitting effect, so it is considered that the fitting parameters under different confining pressure conditions do not change. That is, under the same mix ratio, the fitting parameters for the *D–γ* curve of the soil mixture are the same. Table 4 presents the fitting parameters for the *D–γ* curves of mixtures under different mix ratios. Analysis of the data in the table reveals that as the rubber content increases, $D_{\max}$ shows an increasing trend. Compared to pure clay, the $D_{\max}$ of the mixture with 5% rubber content increases from 2.76% to 7.93%. However, when the rubber content continues to increase, $D_{\max}$ does not show significant growth, meaning that in practice, a mixture with 5% rubber content can be used as the modified clay. On one hand, the damping ratio of the mixture is significantly improved; on the other hand, it reduces the amount of rubber used and lowers construction difficulty. Additionally, as the rubber particle content increases, $D_{\max}$ shows a slight growth, indicating that increasing rubber particles can enhance the damping performance of the mixture. In practical soft soil engineering, large-particle-size (2 mm) rubber particles should be selected as damping materials.

The maximum damping ratio of the CRM is 7.7%, which may appear modest in absolute terms. However, the pure CL clay in this study, due to its high fine-particle content and dense structure, has a native maximum damping ratio of only 2.76%. The incorporation of rubber particles increases this value to 7.7% for the CRM, representing a increase of approximately 179%. For a low-damping material like clay, nearly doubling its damping capacity can substantially improve its ability to dissipate dynamic load energy and resist fatigue under cyclic loading. While this enhancement is accompanied by a reduction in shear modulus, many vibration mitigation or cushioning engineering applications (e.g., subgrade isolation layers) precisely allow for a trade-off where some stiffness is sacrificed to achieve significant damping improvement.

Initially, at a low rubber content (=5%), each added rubber particle creates a new, highly dissipative clay–rubber interface. Frictional slippage at these new interfaces is the dominant damping mechanism, leading to a rapid rise in $D_{\max}$. However, the specific surface area of the clay matrix available for creating effective contact is finite. Beyond a certain threshold, additional rubber particles do not proportionally increase the total active interfacial area available for energy dissipation, as the clay surface becomes increasingly saturated.

Clay-rubber mixtures (CRMs) show strong potential for durable geotechnical applications due to the environmental resilience imparted by the rubber phase. Research on rubber-based engineering components, such as under-sleeper pads, proves that elastomeric materials maintain structural integrity under harsh conditions. For example, after standardized tests involving water, freeze-thaw cycles, and temperature extremes, key stiffness parameters of rubber pads changed by only ≤3.1% [14]. This inherent durability is transferred to the CRM. The rubber particles distributed within the clay matrix similarly exhibit excellent durability. Consequently, its shear modulus and damping ratio are less affected by external environmental conditions. Therefore, CRM is particularly suitable for engineering applications that require long-term exposure to significant climatic and temperature variations, such as sustainable road embankments, vibration damping layers, and landscape fill projects. The combination of improved mechanical properties and proven environmental resistance makes CRM a reliable and sustainable engineering material.

## 4. Conclusions

In this study, GDS resonance column equipment was employed to conduct systematic resonance column tests on clay-rubber mixtures. The focus was on exploring the evolution of the small-strain shear modulus (*G*) and damping ratio (*D*) of clay-rubber mixtures (CRMs) under the influences of stress level ($\sigma_{3}$), rubber particle content ($C_{\mathrm{rubber}}$), and rubber particle size ($D_{\mathrm{rubber}}$). In addition, the evolution of the maximum dynamic shear modulus ($G_{\max}$) of CRM under small-strain conditions was analyzed. The main conclusions are as follows:

1. With the increase in $C_{\mathrm{rubber}}$, the *G* values significantly decrease, while the *D* values increase. This indicates that rubber particles inhibit the development of the small-strain stiffness of CRM and enhance the shock absorption capacity of CRM.
2. With the increase in $D_{\mathrm{rubber}}$, the *G* values slightly increase and the *D* values change minimally. This suggests that the increase in rubber particle size leads to a reduction in the specific area of the rubber particles and the direct contact area between clay particles and rubber particles, resulting in an increase in the *G* values.
3. The *G–γ* curve of CRM was analyzed using the Hardin–Drnevich equation. It was found that the *G–γ* curve of CRM could be well described by this equation and the $G_{\max}$ values with various $C_{\mathrm{rubber}}$ and $D_{\mathrm{rubber}}$ were obtained. According to the evolution of $G_{\max}$, a model considering the influences of $\sigma_{3}$ and $C_{\mathrm{rubber}}$ is proposed. After being verified by the experimental data in this study, it was found that the applicability of this model is good.
4. The *D–γ* curve of CRM was analyzed using an empirical formula. It is obvious that the rubber particles can improve the damping ratio of CRM. The increase in rubber particle size enhances the damping ratio of CRM. Furthermore, for CRM, a 5% rubber particle content already significantly improved its damping performance. From both economic and practical perspectives, selecting “large-diameter rubber particles with $C_{\mathrm{rubber}}$ = 5%” as an additive for clay modification can effectively enhance its damping performance.

## Figures and Tables

**Table 1:** Resonant column test scheme of the clay-rubber mixtures.

Content of Rubber (%)	Diameter of Rubber Particle (mm)	Consolidation Pressure (kPa)	Range of Shear Strain (%)
0, 5, 10, 15	15	50, 100, 200, 300, 400	0.001–0.1
15	0.085, 0.25, 2.0	50, 100, 200, 300, 400	0.001–0.1

**Table 2:** Summary of fitting parameters of *G–γ* curves of CRM at $D_{\mathrm{rubber}}$ = 0.25 mm with different rubber particle contents.

Confining Pressure (kPa)	C_rubber_ = 0%		C_rubber_ = 5%		C_rubber_ = 10%		C_rubber_ = 15%	
	*G*_max_ (MPa)	*γ*_ref_ (%)	*G*_max_ (MPa)	*γ*_ref_ (%)	*G*_max_ (MPa)	*γ*_ref_ (%)	*G*_max_ (MPa)	*γ*_ref_ (%)
50	36.9835	0.1801	32.8877	0.1006	29.6188	0.08986	27.2292	0.08707
100	58.9512	0.1286	50.5757	0.1197	40.9841	0.08716	36.6901	0.09489
200	79.2236	0.1718	68.6427	0.1020	58.2475	0.1228	52.2331	0.07423
300	89.8444	0.2700	79.3730	0.1955	65.2858	0.1604	58.6850	0.1292
400	94.8017	0.3279	85.3447	0.2546	70.7175	0.1951	62.6165	0.1596

**Table 3:** Summary of fitting parameters of *G–γ* curves of CRM at $C_{\mathrm{rubber}}$= 15% with different rubber particle sizes.

Confining Pressure (kPa)	D_rubber_ = 0.085 mm		D_rubber_ = 0.25 mm		D_rubber_ = 2 mm	
	*G*_max_ (MPa)	*γ*_ref_ (%)	*G*_max_ (MPa)	*γ*_ref_ (%)	*G*_max_/MPa	*γ*_ref_ (%)
50	24.5939	0.07619	27.2292	0.08707	27.9593	0.09618
100	38.4871	0.06360	36.6901	0.09489	40.4729	0.1168
200	49.2632	0.06477	52.2331	0.07423	49.5981	0.1315
300	55.3950	0.1185	58.6850	0.1292	64.9591	0.2130
400	60.2564	0.1059	62.6165	0.1596	71.7926	0.1743

**Table 4:** Summary of fitting parameters of *D–γ* curves of CRM with different rubber particle contents and particle sizes.

$C_{\mathrm{rubber}}$ (%)	$D_{\mathrm{rubber}}$ = 0.085 mm			$D_{\mathrm{rubber}}$ = 0.25 mm			$D_{\mathrm{rubber}}$ = 2 mm		
	$D_{\max}$ (%)	$\gamma_{\mathrm{ref}}$ (%)	*n*	$D_{\max}$ (%)	$\gamma_{\mathrm{ref}}$ (%)	*n*	$D_{\max}$ (%)	$\gamma_{\mathrm{ref}}$ (%)	*n*
0	—	—	—	2.7620	0.2157	0.2025	—	—	—
5	—	—	—	7.9323	0.1545	0.4769	—	—	—
10	—	—	—	7.4368	0.1311	0.4263	—	—	—
15	6.7763	0.0858	0.5581	7.7164	0.1090	0.4031	10.4057	0.1464	0.4830

## Data Availability

The original contributions presented in this study are included in the article. Further inquiries can be directed to the corresponding author.

## List of Symbols

*a*, *b*, *c*, d, *m*, *n*, and $\gamma_{0}$	fitting parameters
*e*	pore ratio
*D*	damping ratio
*G*	shear modulus
$C_{\mathrm{rubber}}$	content of the rubber particles
$D_{\mathrm{rubber}}$	diameter of the rubber particles
$D_{\max}$	maximum damping ratio
$G_{\max}$	maximum dynamic shear modulus
$e_{\mathrm{eq}}$	equivalent pore ratio
$\gamma_{\mathrm{ref}}$	reference shear strain
$\sigma_{3}$	stress level
$V_{\mathrm{void}}$	pore volume of clay
$V_{\mathrm{rubber}}$	volume of rubber particles
$V_{\mathrm{clay}}$	volume of clay particles
